# Current status and reflections on fertility preservation in China

**DOI:** 10.1007/s10815-022-02648-0

**Published:** 2022-11-02

**Authors:** Jiakai Zhang, Lun Wei, Xiaoling Deng, Chao Luo, Qianmeng Zhu, Shucheng Lu, Caiping Mao

**Affiliations:** 1 Reproductive Medicine Center, First Affiliated Hospital of Soochow University, 899 Pinghai Rd, Suzhou Suzhou, Jiangsu, 215000 China; 2 Marxism Research Institute, Soochow University, Suzhou, Jiangsu, 215123 China; 3 Suzhou High School Affiliated to Xi’an Jiaotong University, Suzhou, Jiangsu, 215000 China

**Keywords:** Fertility preservation, China, Application scope, Posthumous-assisted reproduction, Compassionate preservation, Reproductive ethics

## Abstract

**Purpose:**

With the progress of medical technology and renovated conception of fertility, the prospective studies and practice of fertility preservation are drawing more and more attention from medical workers. With the largest population of over 1.4 billion, China makes the experience accumulated in fertility preservation efforts even more relevant. This article summarizes China’s experience and shares it with the world to promote the healthy development of fertility preservation.

**Methods:**

This study was based on multiple Chinese expert consensuses on fertility preservation issued in 2021 and the current national regulations and principles, compared with the latest advice and guidelines issued by global reproductive authorities such as the ASRM and ESHRE. Summarize the experience and reflection of Chinese scholars in the process of fertility preservation.

**Results:**

This study reports on the current situation of fertility preservation in China, sharing the Chinese experience gained in the process of development, and offering Chinese reflections on worrying issues.

**Conclusion:**

Fertility preservation is a medical and social issue of reproductive health security, which is conducive to the sound development of the world population and social production.

## Introduction

As fertility rates are declining globally, ART has been designed to address the fertility needs of couples with infertility since the very beginning, and as medical technology advances and ideology on childbearing changes, fertility protection and preservation based on prospective fertility appeal are becoming increasingly important to ART practitioners and reproductive biologists. Since the first fertility preservation guidelines were published by American Society of Clinical Oncology in 2006 [1], fertility preservation has been practiced on a global scale. In 2021, China published the Chinese Expert Consensus on Fertility Preservation [2] and the Chinese Expert Consensus on Male and Female Fertility Preservation [3, 4], but China’s work is still in its infancy.

Starting from the current status of fertility preservation in China and with reference to the latest recommendations and guidelines published by global professional authorities such as ASRM, SART, ESHRE, and ISFP, this article discusses China’s decisions and reflections on the development of fertility preservation, summarizes China’s experience, and shares it with the world to promote the healthy development of fertility preservation.

## The current situation of fertility in China

Population issues are increasingly attracting the attention of the international community, and the birth rate in China was 8.52‰ in 2020, falling below 10‰ for the first time, while the natural population growth rate was only 1.45‰ in the same period [5]; the total fertility rate of 1.3 was lower than the international warning line of 1.5, a record low [6]. The average age of first childbearing for Chinese women was postponed from 24.3 years to 27.3 years from 2006 to 2017 [7], which has caused a superimposed effect on the decline of fertility. The Chinese government fully implemented the two-child and three-child fertility policies in 2015 and 2021, respectively, incorporating the promotion of population fertility into the basic state policy [8]. However, this has been followed by a significant increase in the number of pregnant women of advanced maternal age; according to statistics, in 2016, there were about 90 million couples eligible for the two-child policy in China, 60% were above 35 years of age and 50% were above 40 years of age, and the risk of maternal comorbidities and complications as well as birth defects will be greatly increased [9].

In addition, the incidence of infertility/sterility worldwide shows an increasing trend, which has become a global medical and social problem. There is a lack of national large-scale data on the prevalence rate of infertility/sterility in China. In regional studies, the prevalence rate of infertility among women of reproductive age varies, but still shows an overall increasing trend. According to recent reports, the prevalence rate of infertility among women of childbearing age in Henan and Liaoning provinces of China reached 24.6% and 13.4%, respectively, in 2021 [10, 11]. The most direct way to assess male infertility is to examine the parameters of semen. And sperm concentration in fertile men in China decreased significantly from 1984 to 2019, from 98.85 × 10^6^/ml to 72.55 × 10^6^/ml [12]; the human sperm bank in Hunan Province, China, counted the quality of sperm donated by more than 30,000 young men from 2001 to 2015, and in addition to a significant decrease in the sperm concentration (the number of sperm per milliliter of semen), the percentage of sperm with normal morphology decreased from 31.8 to 10.8%, and the progressively motile sperm count decreased from 34 × 10^6^/ml to 21 × 10^6^/ml [13].

In summary, China’s population growth rate and fertility rate are decreasing year by year, the trend of delaying the childbearing age is serious, and the prevalence rate of infertility/sterility is increasing year by year, which has a long-lasting and far-reaching impact on social production and economic development. It is imperative to implement measures to protect and preserve the fertility of Chinese men and women of childbearing age as soon as possible and to promote long-term balanced population development.

## Current status of fertility preservation in China

**Fig. 1:**
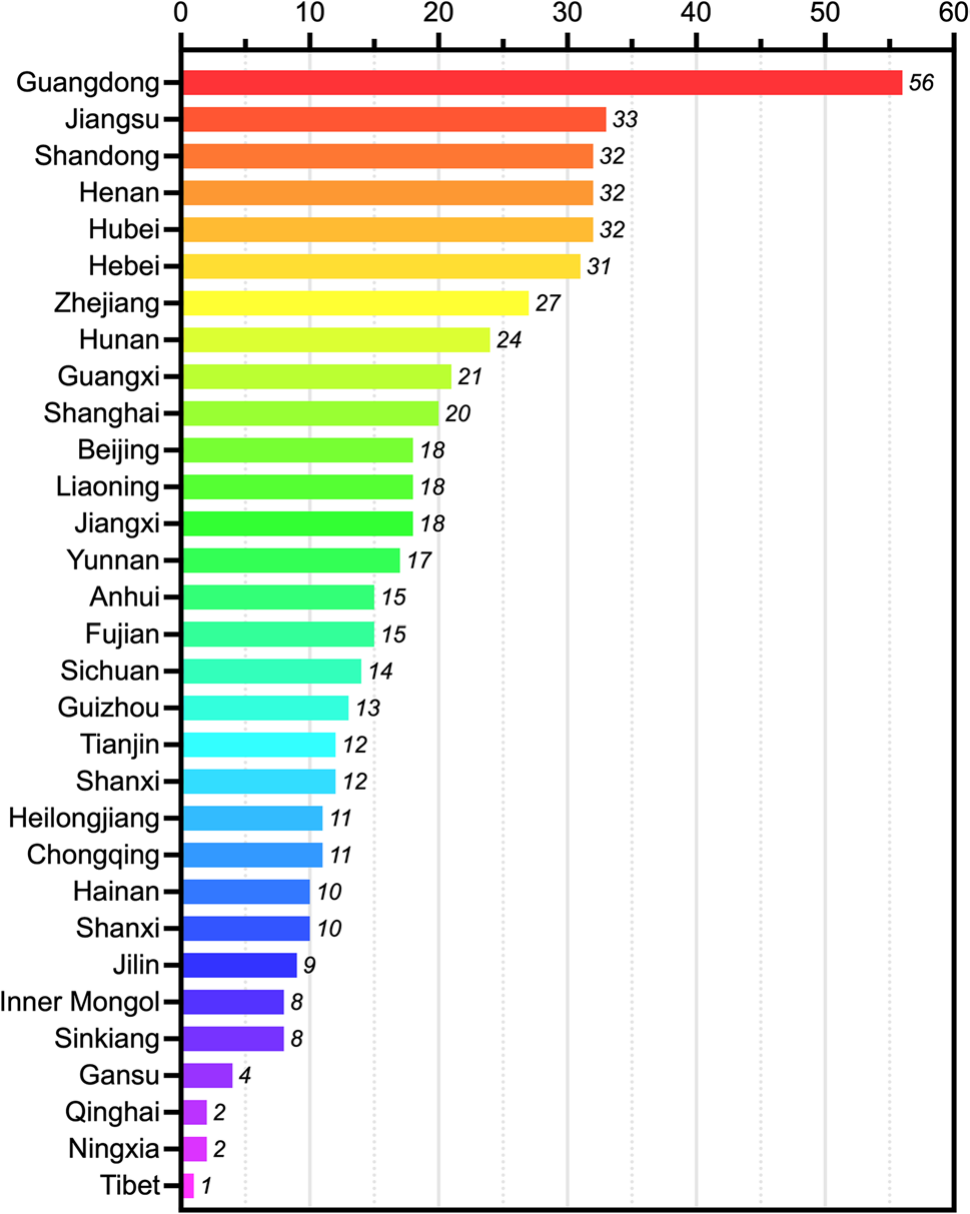
Statistics on the number and regional distribution of ART medical institutions in mainland China

At present, China lacks special statistics on fertility preservation, so this section will indirectly reflect the current situation of fertility preservation in China through the existing data. As of December 2020, there were 536 medical institutions and 27 human sperm banks approved to carry out ART in Chinese mainland. Guangdong has 56 ART medical institutions, ranking first among mainland provinces. Then there are 33 in Jiangsu, and 32 in Shandong and Henan, respectively. However, there is only 1 ART medical institution in Tibet, followed by 2 in Qinghai and Ningxia, respectively (Fig. 1) [14]. In summary, the allocation of ART medical institutions in China has increased year by year, and the availability of technical services has improved significantly, but the development among regions is unbalanced.

**Fig. 2:**
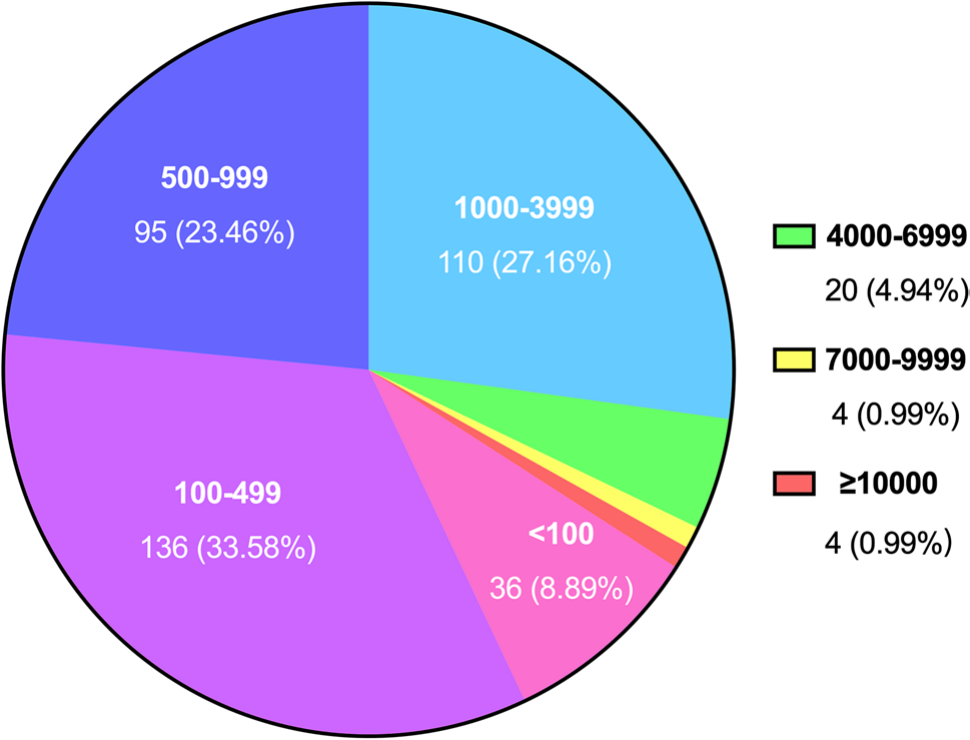
Statistics on the number of oocyte retrieval cycles in mainland China

The number of oocyte retrieval cycles can directly reflect the scale and volume of ART medical institutions. According to the data published at the CSRM Annual Meeting in August 2022, there are 4 mainland ART medical institutions with 10,000 cycles or more (accounting for 1.0%) and there are 24 institutions with more than 4000 cycles (accounting for 5.9%) in 2020. 341 institutions with cycles ranging from 100 to 4,000 are the majority (accounting for 84.3%), but there are 36 institutions with less than 100 cycles (accounting for 8.9%) (Fig. 2) [15]. From the above, it can be seen that China’s ART medical institutions are large in scale and volume, and their service capacity is constantly improving, but it also highlights the problem of uneven development among institutions.

**Fig. 3:**
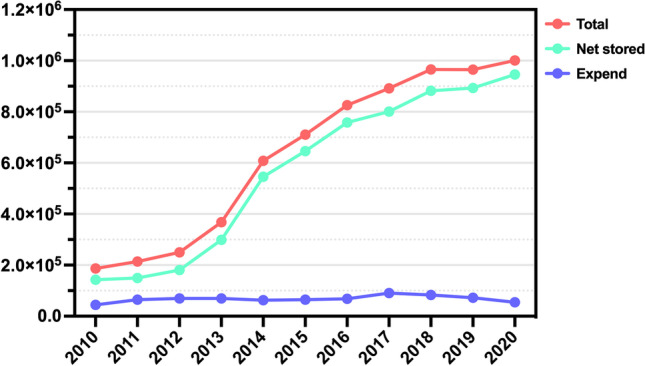
Statistical data of human sperm bank in mainland China from 2010 to 2020

According to the published data of China’s human sperm bank, the total number of stocks increased from 187 thousand in 2010 to 1 million in 2020. In 10 years, the number of net stored sperm increased from 143 to 946 thousand, becoming 6 times more (Fig. 3) [15]. It shows that the service ability of Chinese human sperm bank is improved, which can meet the clinical needs of male fertility preservation. Although the continuous development of China’s sperm bank has brought us good news, this problem is more prominent and urgent for women with fertility preservation needs. This also puts forward higher requirements and greater challenges to China’s fertility preservation work.

## Application scope of fertility preservation in China

Fertility preservation begins with the forward-looking protection and preservation of fertility in patients with diseases that predictably affect fertility, or medically induced injuries that inevitably disrupt reproductive function during the course of treatment. A clear scope of application is a prerequisite for the standardization of fertility preservation, and a comparison of national attitudes toward preservation in different populations illustrates the current status of fertility preservation in China (Table 1).

**Table 1:** Compare the application scope of fertility preservation in China with that of ASRM and ESHRE

	Cancer patients		Non-cancer patients		Healthy adult		Child		Special population
	♀	♂	♀	♂	♀	♂	♀	♂	
ASRM	√	√	√	√	√	√	√	√	√
ESHRE	√	√	√	√	-	√	-	√	-
China	√	√	√	√	×	√	-	√	×

√ means applicable; X means not applicable;—means cannot be identified

### Cancer/benign conditions patients

With the rapid development of medical technology, the survival rate of cancer patients has increased significantly. According to the latest data from the National Center for Health Statistics, cancer mortality rates have continued to decline since 1991, with an overall decrease of 29% and approximately 2.9 million cancer patients surviving [16]. Meanwhile, the incidence of cancer in AYA increased by nearly 30% from 2011–2015 compared to 1975–1979 [17]. The trend of younger and younger incidence rates leads to more and more cancer patients having reproductive needs, and fertility preservation before starting a treatment cycle can be the best option to meet their fertility appeal.

In the Chinese Expert Consensus on Male Fertility Preservation, recommendations were classified into three levels 4 (Table 2). In the Chinese Expert Consensus on Female Fertility Preservation, the assessment of the risk of ovarian impairment is used as the main basis for clarifying the scope of application [4, 18]. It mainly includes oncologic diseases with the most significant trend of rejuvenation, such as breast cancer, gynecologic tumors, and hematologic tumors, and also non-oncologic diseases with a high risk of fertility decline, such as autoimmune diseases, endometriosis, and Turner syndrome. The consensus elaborates on the impact of different diseases, various stages, and treatment strategies on fertility, and proposes corresponding fertility preservation measures and recommendations.

**Table 2:** Scope of application and recommended grades of male fertility preservation in china

Recommended grades	Application scope
A	Postpuberal and adult male cancer patients
B	Prepuberal male cancer patients
	Patients who had difficulty in obtaining sperm, or were unable to obtain sperm on the day of ART due to personal factors, or required surgery to obtain sperm
	Other male with fertility preservation needs
C	Patients with autoimmune diseases that affect male fertility

A means highly recommended (the evidence is positive, can improve health outcomes, the benefits outweigh the disadvantages); B means recommended (there is good evidence that can improve health outcomes, the benefits outweigh the disadvantages); C means not routinely recommended, optional (evidence exists to improve health outcomes, but risk–benefit ratios cannot be identified)

With the concerted efforts of multidisciplinary physicians and scientists in reproductive medicine and oncology worldwide, optimal fertility preservation strategies are now available for cancer and benign conditions patients of all types and stages, which can maximize the protection and preservation of their fertility. There is a worldwide consensus on the significance and effectiveness of fertility preservation before foreseeable reproductive impairment occurs [19–21].

### Healthy adults

Ageing as an independent risk factor affecting fertility makes fertility preservation in healthy adults feasible from a reproductive health and preservation technology perspective, but this is not uniformly understood and policy-wise across countries [20, 22]. Men have a larger scope and a higher degree of tolerance in determining the applicability of fertility preservation due to differences in technical difficulty and social ethical constraints in fertility preservation by gender.

The Chinese Expert Consensus on Male Fertility Preservation clearly states that all men with fertility preservation needs have the right to fertility preservation and rates such appeal as level B which is recommending fertility preservation [3]. And the Chinese Expert Consensus on Female Fertility Preservation does not mention fertility preservation recommendations for healthy adult women [4]. However, under current policy in mainland China, egg freezing can only be performed on married women with indications. The guidelines issued by organizations such as ASRM and ESHRE emphasize risk-informed consent and respect for reproductive rights [19, 20] and are more positive than China’s guidelines on the issue concerning whether fertility preservation can be performed on healthy adults. Moreover, in some countries or continents, governments have allowed the commercialization of “sperm banks” and “egg banks.”

### Children

According to the Chinese Expert Consensus on Male Fertility Preservation, fertility preservation in underage males should be performed with the consent of their guardians after a thorough evaluation by oncologists and reproductive medicine physicians [3]. According to the Chinese Expert Consensus on Female Fertility Preservation merely mentions the fertility preservation strategy of ovarian tissue freezing for underage oncology patients [4, 18]. Fertility preservation in children is performed internationally with equal caution.

At the technical level, the techniques for obtaining and freezing sperm and eggs are relatively mature, while reproductive tissue freezing is still in the experimental stage for adolescents and young adults with immature or underdeveloped sexual development [23]. Regardless of gender, these methods involve invasive operations and still have a long way to go for reproductive tissue freezing techniques due to the uncertain risk of tissue contamination for malignant tumors, blood disorders, etc. [24]. However, experts and scholars from various countries have given full recognition to the development of counseling for fertility preservation in children, emphasizing the importance of pediatricians’ inclusion in pre-treatment recommendations, and pointing out that fertility preservation in adolescents and young adults is still at the counseling and recommendation stage, which is difficult to implement universally due to the lack of technical support and supporting management systems for them [25–28].

### Groups requiring special attention

According to the current law of China, it is forbidden for medical personnel to implement ART for single women and couples who do not meet the national population and family planning laws and regulations. In addition to the above specific groups, there are various “non-standard” situations and relationships with fertility needs, including but not limited to single women, unmarried couples, the unmarried, homosexuals, transgender people, and people with high-risk occupations. With the development of economy, social is becoming more and more diversified, and the proportion of special populations is increasing. The achievements of the human assisted reproduction and fertility preservation technologies have become the primary, or even the only, way for this population to exercise their reproductive rights. None of the Chinese expert consensus mentions fertility preservation recommendations for the above-mentioned special populations [2–4]. According to current Chinese regulations, fertility centers are temporarily unable to perform fertility preservation services for this population for the reasons mentioned above.

In recent years, developed countries have taken a more positive view of fertility preservation in this groups. High-risk occupational groups, such as front-line military personnel, military police, firefighters, pilots, and nuclear-radiated close contacts, whose occupational activities pose a high risk to life safety, should be made more aware of fertility preservation techniques, and early completion of fertility protection and preservation is recommended. In general, fertility preservation claims should be safeguarded for all types of populations, except in cases where there is clear evidence-based medical evidence of serious life and health risks to future generations [19, 20]. It is undeniable that a high degree of social acceptance, tolerance, and a well-established sperm/egg bank management system can provide more comprehensive and professional medical services for all types of people with fertility preservation needs.

The expert consensus of organizations such as ASRM and ESHRE has a more positive attitude toward the preservation of reproduction among sexual minorities. It is emphasized that categorically denying the reproductive needs of sexual minorities is disrespectful and a violation of human rights [29, 30]. Attitudes toward sexual minorities are closely related to national marriage laws, and since 2000, when the Netherlands was the first country in the world to legalize same-sex marriage, a total of 37 countries and territories have legalized same-sex marriage by the end of 2020. Taiwan became the first region in Asia to recognize same-sex marriage in 2017, which provides a research base and legal guarantee for China to carry out fertility preservation work for sexual minorities.

## Reflections on fertility preservation in China

Although fertility preservation was introduced late in China, it has developed rapidly. The world’s first case of live birth after autologous transplantation of frozen ovarian tissue was reported in Belgium in 2004 [31], and China also successfully broke through the technology of live birth after transplantation in 2017 [32], and more than 200 babies have been born worldwide by applying this technology so far [33]. Medical technology is developing rapidly, but the transformation and application of scientific and technological achievements involves many realistic factors such as cultural background, social environment, management level, and ethical issues. Technology not only has the positive value of meeting human needs but also will bring certain crises, hidden dangers, and even catastrophic negative effects. This section will discuss the concerns and considerations in the process of fertility preservation in China, with the aim of promoting the healthy development of fertility preservation in the world.

### Technology options and risks

**Fig. 4:**
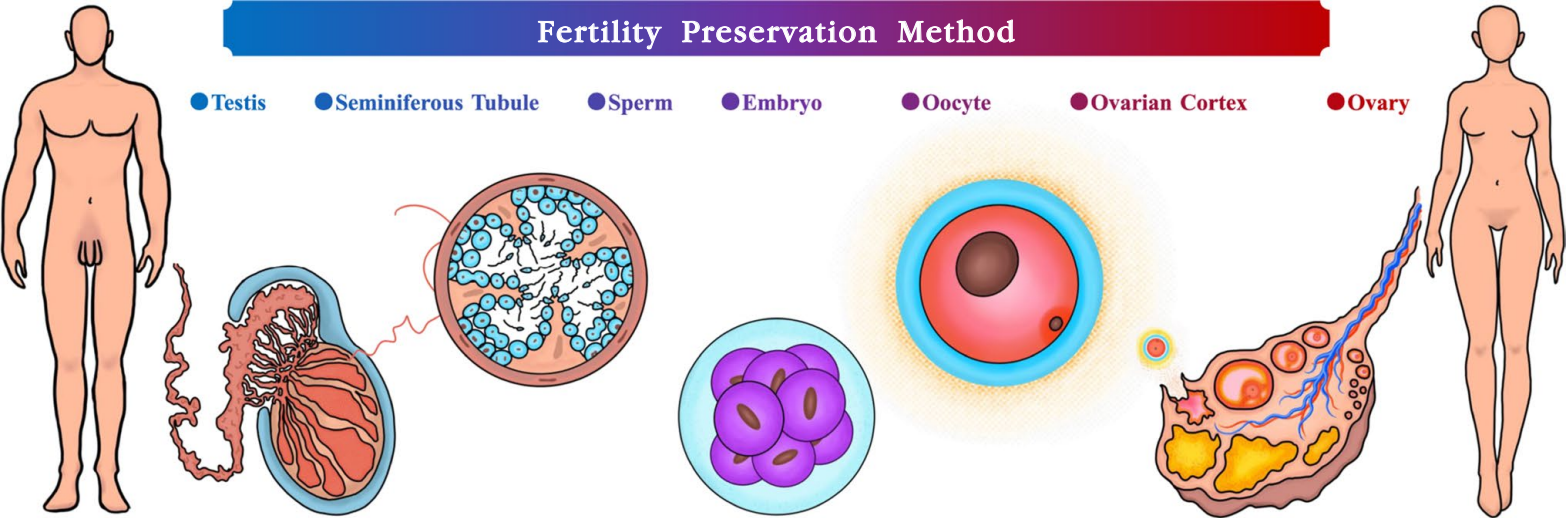
Gametes/embryos/tissues that can be used for fertility preservation

In the implementation of fertility preservation, it is important to select and precisely apply the appropriate fertility preservation method for each patient in order to achieve precise medical services that minimize medical damage, minimize medical costs, and maximize patient benefits. Based on basic research results in reproductive biology and cryobiology, the available fertility preservation technologies include embryo freezing, gamete (sperm/oocyte) freezing, reproductive tissue freezing, and sub-tissue freezing (Fig. 4), with varying levels of basic research and clinical application, which highlights the expertise and importance of the fertility physician in making decisions.

Ovarian/testicular tissue cryopreservation of patients, one of the most prominent clients in fertility preservation efforts, will inevitably carry residual cancer cells, posing a risk of malignancy recurrence for future in situ transplantation. Although rare, there is still a greater risk of recurrence for certain tumors with a high risk of recurrence [34]. For certain types of hematologic tumors, there are individual differences in the damage of radiotherapy and chemotherapy on fertility, and data show that about 50% of patients still have a chance of natural pregnancy in the future, and how to avoid excessive medical interventions should be given full consideration [35]. In addition, the use of ovulation-promoting drugs to obtain more oocytes in practice, especially for reproductive/endocrine system tumors, will likely have the undesirable consequence of stimulating tumor cell growth or delaying the timing of tumor treatment [36]. It would be putting the cart before the horse to compromise the effectiveness of tumor treatment or even reduce patient survival due to fertility preservation.

Cryobiology is the core foundation of fertility preservation technology, and we have experienced the progress from slow freezing to cryopreservation. Currently, vitrification is used as the major method of cryopreservation. Although milestones have been achieved in its development, it is still inconclusive whether it will pose long-term effects over time. The use of frozen embryo transfer, this cohort study includes slow freezing and cryopreservation, was significantly associated with an increased risk of cancer in children, mainly including leukemia and neurological tumors [37]. In 2020, the European Cancer Research Center also clarified that frozen embryo transfer was associated with an increased incidence of cancer in offspring [38]. Professor Huang Hefeng, a member of the Chinese Academy of Sciences, has long been concerned about the safety and genetic effects of human ART, and a recent study by this team found that male mouse offspring transferred after freezing–thawing showed impaired glucose metabolism, mainly manifesting as insulin resistance [39]. In addition, correlations with the risk of high birth weight, neurological disorders, metabolic disorders, and cardiovascular diseases have been reported [40–43]. Because of the insidious and cumulative effects of cell cryopreservation on offspring, continued and more in-depth studies are needed.

### Construction of human “egg bank”

In China, there is still no national plan for the establishment of “egg banks,” and only some qualified fertility centers have set up “egg freezing and storage banks” on an exploratory basis. Throughout the world, the first human egg bank was established in Argentina in 2002, and in 2013, the ASRM issued guidelines that no longer considered oocyte freezing a research project, but as a routine ART technique [44]. Since then, national academic organizations in reproductive medicine have issued consensus and guidelines to promote the development of “egg banks” [19, 34].

Compared with women, the medical technology, the high volume of male spermatozoa per discharge, the non-invasive sperm acquisition, the mature technology of sperm freezing and resuscitation, the social background, the high acceptance of the public, the low ethical controversy, and the low space for commercialization, have laid a good foundation for the pioneering work of male fertility protection and the establishment of “sperm bank.” China’s first human sperm bank was established in 1981, and after more than 40 years of development, China’s human sperm banks have formed a relatively standardized and perfect management system to ensure the smooth development of male fertility preservation in China.

The most influential journal in the field of reproductive medicine in China published a special feature in 2022, highlighting the medical and social importance of establishing “egg banks” in China [45], but at the same time raising several practical issues that should alert relevant practitioners. First, it potentially alters women’s fertility patterns and induces women to give up their fertility opportunities at an appropriate age, increasing the risk of high-risk pregnancies and perinatal complications. Second, the extent to which donors should be quarantined and screened, especially for carriers of genetic diseases, can vary from country to country depending on medical conditions, laws, and cultures. Finally, hot topics such as the potential risk of egg quality, whether the age of the recipient should be limited, and financial compensation for donors are reviewed. It is pointed out that adequate research and evidence should be conducted, and the principles of stepwise, pilot, and planning can be used to promote technical improvement, strengthen regulation, and carry out fertility preservation in a reasonable and healthy manner.

### Posthumous-assisted reproduction

PAR is a special phenomenon in the development of ART, including both planned PAR and unplanned PAR—planned PAR means that the deceased had fertility preservation during his or her lifetime, i.e. there was already frozen sperm, eggs, or embryos; unplanned PAR is a sudden accident or death due to an acute illness occurs to the deceased, and his or her spouse or parents request gametes from his or her remains for ART [46]. The latest ASRM Consensus on PAR states that if the deceased did not sign the relevant written documents before death, the spouse or parents may request ART after the written request [47].

The first PAR case was publicly reported in China in 2001, which occurred in the case of Zheng Xueli in Zhejiang Province, where the court denied the client’s request for postmortem sperm retrieval for PAR from the husband of the condemned prisoner [48]. The first planned PAR occurred in 2004 in the case of Wang Xia in Guangdong Province, where the client’s husband intended to use frozen embryos for delivery after his accidental death in a car accident, and the court eventually granted his request to continue using frozen embryos [49]. The first unplanned PAR occurred in 2005 in Taiwan in the case of Sun Jixiang, whose family and girlfriend wanted to perform a PAR after his death in the line of duty and were immediately rejected by the court. Subsequent cases of unplanned PAR were not followed by IVF to produce PAR offspring [49].

In Chinese judicial practice, there is an increasing emphasis on the “Best Interests of the Child” as an important theoretical support for adjudication. For PAR offspring, it is inevitable that they will have the single-parent or reconstituted family as a special growing environment with the psychological and mental aspects of the possible irreversible harm, which is unfair to the offspring and irresponsible of the court decision. At the same time, how to identify the legal status of PAR offspring and protect their rights and interests needs to be urgently resolved. Moreover, PAR inevitably violates the dignity of the deceased and infringes on the deceased’s right to informed consent; and the remains and tissues of the deceased do not belong to general objects and are not legal objects, so they cannot be disposed of by their relatives and friends as the subject of inheritance.

In China, due to the deep-rooted traditional concept of “carry on the family line,” especially after the accidental death of a male in a family, his parents and spouse will have an extremely strong claim to the PAR. In addition to considering the interests of the PAR applicants, the rights and interests of the deceased should be fully respected, and the interests of the PAR descendants should be fully evaluated. Countries with similar cultural backgrounds and social environments should be equally cautious when conducting PAR. This not only poses new challenges to medical technology, laws, and regulations, but also places higher demands on civilization.

### Compassionate preservation

A recommendation published by the ASRM is worth noting. A patient has strongly requested that her physician transfer embryos into her body when conception is almost impossible. The ASRM believes that there are valid and reasonable reasons to support the patient’s decision to transfer at his or her own discretion in order to safeguard his or her reproductive right, and defines this type of embryo transfer as “compassionate transfer” [50]. Similarly, we propose the concept of “compassionate preservation” in cases where fertility preservation is not recommended at this stage of research, in patients where fertility preservation does not significantly improve reproductive outcomes, and in cases where there is evidence of serious life and health risks to offspring. For example, ovarian cancer, testicular cancer, metastatic carcinoma from other tissues. For example, ovarian cancer, testicular cancer, metastatic carcinoma from other tissues, at the present level of medical technology, although these preserved reproductive tissues cannot be safely transplanted back and restoring fertility. The psychological treatment is aimed at replacing “abandonment of treatment” with “ineffective treatment,” and aims to achieve a certain degree of “placebo effect” and “hope.” The aim is to achieve a certain degree of “placebo effect” and “hope giving” medical help.

## Perspectives on fertility preservation technologies

**Fig. 5:**
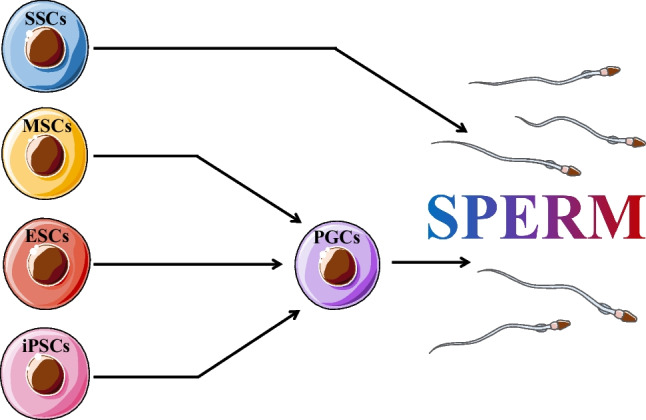
Various stem cells that can be induced to produce sperm. Spermatagonial stem cells, SSCs; mesenchymal stromal cells, MSCs; embryonic stem cells, ESCs; induced pluripotent stem cells, iPSCs; primordial germ cells, PGCs

Stem cells have the ability to self-renew, proliferate, and differentiate into a variety of functional cells with directional differentiation. As the researches of stem cells continue to advance, fertility preservation is gradually explored and practiced, and various stem cells have been found to induce spermatogenesis, including SSCs, MSCs, ESCs, and iPSCs (Fig. 5).

SSCs can be isolated from testicular tissue for cryopreservation and can be differentiated into spermatozoa by autologous transplantation or in vitro culture when the time is right [51–54]. MSCs are derived from umbilical cords, bone marrow, adipose, and systemic vascularized tissues and can differentiate to germ cell lineage in vitro [55–57]. ESCs need to be isolated from proto-intestinal pre-embryonic or primordial gonads, so research stays in animal experiments [58, 59]. Human iPSCs technology can be used to obtain PGCs by inducing somatic cells to express a specific gene, which can then be implanted in vivo to induce normal reproductive physiological processes [60–62]. Because oogenesis is more complex, studies on in vitro differentiation of stem cells or improvement of the ovarian microenvironment have only been conducted in animal studies [63, 64]. Although stem cell technology is in the exploratory stage in the field of fertility protection, its clinical significance and application prospects are very much worthy of recognition and expectation.

With the support of national policies and increasing awareness of ART, and with the growing social demand for fertility preservation, fertility preservation in China has much room for development. Harboring the aim of promoting China’s progress together with the rest of the world and contributing China’s strength and wisdom to human fertility preservation, we actively learn about key issues in fertility preservation, discuss hot topics that have attracted widespread social attention, share China’s experiences and solutions, and present China’s concerns and thoughts.

## Abbreviations

ART: Assisted reproductive technology
ASRM: American Society for Reproductive Medicine
ESHRE: European Society of Human Reproduction and Embryology
SART: Society for Assisted Reproductive Technology
ISFP: International Society of Fertility Preservation
CSRM: Chinese Society of Reproductive Medicine
AYA: Adolescents and young adults
PAR: Posthumous-assisted reproduction
IVF: In vitro fertilization
SSCs: Spermatagonial stem cells
MSCs: Mesenchymal stromal cells
ESCs: Embryonic stem cells
iPSCs: Induced Pluripotent Stem Cells
PGCs: Primordial germ cells
